# Pharyngeal microbiome alterations during *Neisseria gonorrhoeae* infection

**DOI:** 10.1371/journal.pone.0227985

**Published:** 2020-01-16

**Authors:** Antonella Marangoni, Camilla Ceccarani, Tania Camboni, Clarissa Consolandi, Claudio Foschi, Melissa Salvo, Valeria Gaspari, Antonietta D’Antuono, Matteo Belletti, Maria Carla Re, Marco Severgnini

**Affiliations:** 1 Microbiology, DIMES, University of Bologna, Bologna, Italy; 2 Institute of Biomedical Technologies–National Research Council, Segrate, Milan, Italy; 3 Department of Health Sciences, University of Milan, Milan, Italy; 4 Dermatology, St. Orsola-Malpighi Hospital, Bologna, Italy; University of Minnesota Twin Cities, UNITED STATES

## Abstract

Pharyngeal gonorrhoea is a common sexually transmitted infection among ‘men having sex with other men’ (MSM). *Neisseria gonorrhoeae* (NG) pharyngeal infections are usually characterized by the absence of symptoms, acting as an important reservoir for their further spread. To the best of our knowledge, no information about the composition of the pharyngeal microbiome during an ongoing NG infection is currently available. Therefore, in this study, we characterized the pharyngeal bacterial community profiles associated with NG infection in a well-selected cohort of HIV-negative MSM reporting unsafe oral intercourse. A total of 70 pharyngeal swabs were considered, comparing non-infected subjects (n = 45) versus patients with pharyngeal gonorrhoea (n = 25) whose microbiota composition was analyzed from pharyngeal swabs through sequencing of hypervariable V3-V4 regions of the 16S rRNA gene. The pharyngeal microbiome of all subjects was dominated by *Prevotellaceae*, *Veillonellaceae* and *Streptococcaceae* families. Patients with pharyngeal gonorrhoea harboured a pharyngeal microbiome quite similar to negative subjects. Nevertheless, when looking to less-represented bacterial species (relative abundance approximately 1% or less), an imbalance between aerobe and anaerobe microorganisms was observed in NG-infected patients. In particular, the pharyngeal microbiome of NG-positive individuals was richer in several anaerobes (e.g. *Treponema*, *Parvimonas*, *Peptococcus*, *Catonella*, *Filifactor*) and poorer in various aerobe genera (i.e. *Pseudomonas*, *Escherichia*), compared to non-infected controls. No significant differences were noticed in the distribution of commensal *Neisseria* species of the oropharynx between NG-positive and negative subjects. Metabolic variations induced by changes in the microbiome abundance were assessed by a functional prediction of the bacterial metabolic pathways: a more abundant involvement of D-glutamine and D-glutamate metabolism, carbohydrate metabolism, as well as a greater activation of the energy metabolism was observed in patients with pharyngeal gonorrhoea compared to non-infected individuals. Information about the bacterial composition of the pharyngeal microbiome in case of gonorrhoea could shed light on the pathogenesis of the infection and open new perspectives for the prevention and control of this condition.

## Introduction

Sexually transmitted pharyngeal infections due to *Neisseria gonorrhoeae* (NG) are frequent among ‘men having sex with other men’ (MSM) [1, 2]. NG pharyngeal infections are usually characterized by the absence of symptoms, remaining undetected and untreated and, therefore, acting as an important reservoir for the further spread of the infection [3, 4].

The oropharynx represents a pivotal site for *N*. *gonorrhoeae* residency [5]: it is a suitable ecological niche for NG to replicate and persist over time [6]. Moreover, commensal *Neisseria* species found in this site act as a significant reservoir for genetic material, conferring cephalosporin resistance in *N*. *gonorrhoeae*, and, thus, potentially, leading to the emergence of multi-drug resistant gonorrhoea [7, 8].

The implementation of effective strategies to prevent and treat pharyngeal gonorrhoea is fundamental for reducing the incidence of the infection and the alarming problem of antimicrobial resistance [9, 10].

Currently, there are no studies describing the pharyngeal microbiome during *N*. *gonorrhoeae* infections of the oropharynx trait. In this context, our study aimed to assess, for the first time, the bacterial community profiles of the pharyngeal microbiome associated with NG infection in a cohort of MSM.

Information about the bacterial composition of the pharyngeal mucosa undergoing NG infection could help elucidate the pathogenesis of this condition and could open new perspectives for the prevention, control and treatment of NG pharyngeal infections.

## Materials and methods

### Study population and sample collection

Patients eligible for the study were selected from a group of Caucasian MSM attending the STI Outpatients Clinic of S. Orsola-Malpighi Hospital in Bologna (Italy) and reporting unsafe oro-genital intercourse. Exclusion criteria were: being under the age of 18 years; having used any antibiotic, mouthwashes and xylitol-containing gums in the month preceding the study; suffering from infectious/inflammatory oral pathologies (e.g. oral candidiasis, oral lichen planus); being heavy smokers (>15 cigarettes/day); having a binge consumption of alcohol; being positive for HIV; being positive for pharyngeal *Chlamydia trachomatis* infection; receiving systemic cancer chemotherapy or immunomodulating agents; suffering from uncontrolled thyroid disease or diabetes; reporting clinical symptoms suggestive of periodontal disease (gingival redness, swelling or bleeding). A total of 91 MSM were enrolled.

After a clinical examination, a pharyngeal swab (E-Swab, Copan, Brescia, Italy) for the molecular detection of *C*. *trachomatis* and *N*. *gonorrhoeae* was collected from each patient. On the basis of microbiological results, eligible patients were allocated in one of the following groups: ‘no infection’ (negativity for pharyngeal NG, n = 57) and ‘NG’ (positivity for pharyngeal NG, n = 34). All the patients with NG pharyngeal infection denied the presence of oro-pharyngeal symptoms (e.g. pharyngeal pain, hoarseness).

The study protocol was approved by the Ethical Committee of St. Orsola-Malpighi Hospital (78/2017/U/Tess) and written informed consent to the work was collected from each subject.

### Diagnosis of *N*. *gonorrhoeae* pharyngeal infections

Pharyngeal swabs were processed by Versant CT/GC DNA 1.0 Assay (Siemens Healthcare Diagnostics, Tarrytown, NY, USA), a commercial duplex real-time PCR test simultaneously detecting the presence of *C*. *trachomatis* and/or *N*. *gonorrhoeae* DNA [11]. This molecular assay proved to be extremely sensitive in the detection of extra-genital *N*. *gonorrhoeae* infections (limit of detection: 1.0 copies/mL), with an excellent specificity (no false positive results due to the presence of non-gonococcal *Neisseria* species) [11].

### Analysis of the pharyngeal microbiota

Hypervariable V3-V4 regions of the bacterial 16S rRNA gene from pharyngeal swab genomic DNA were amplified, starting from the remaining part of Versant PCR plate DNA eluate. This approach has been previously and successfully used for the analysis of human microbiome [12].

Indexed libraries were prepared by equimolar pooling (4 nmol/L) and sequenced on an Illumina MiSeq platform with a 2×300 bp run, according to manufacturer’s instructions (Illumina, San Diego, CA, USA).

The 16S rRNA raw sequences were merged using Pandaseq [13], and low quality reads (i.e.: showing stretches of bases with a Q-score <3 for more than 25% of their length) were filtered out and discarded. Bioinformatic analyses were conducted using the QIIME pipeline [release 1.8.0; 14], clustering filtered reads into Operational Taxonomic Unit (OTUs) at 97% identity level. Taxonomic assignment was performed via the RDP classifier [15] against the SILVA database (release 132, https://www.arb-silva.de/fileadmin/silva_databases/qiime/Silva_132_release.zip), with a 0.5 identity threshold. In order to compensate for different sequencing depths, all samples were rarefied to 36800 reads, corresponding to the least sequenced sample.

Bacterial biodiversity and distribution were characterized via alpha- and beta-diversity evaluations. Alpha-diversity was measured through the QIIME pipeline using Chao1, observed species, Faith’s phylogenetic index (PD_whole_tree) and Shannon diversity metrics. Weighted and unweighted UniFrac distances and a PERMANOVA test (“adonis” function) from the R package vegan [version 2.0–10; 16] were used to compare the microbial community structure in beta-diversity analysis.

To investigate metabolic variations induced by changes in the microbiome, a functional prediction of the bacterial metabolic pathways was performed using PICRUSt (Phylogenetic Investigation of Communities by Reconstruction of Unobserved States) software [v 1.0.1, 17] and KEGG pathways database [18, 19].

Species-level classification of *Neisseria* spp. was performed by a BLAST-based re-alignment of all reads classified in the genus to a custom reference database made of all available reference sequences in NIH-NCBI database (ftp://ftp.ncbi.nlm.nih.gov/genomes/refseq/bacteria/) of 12 *Neisseria* species commonly found in the pharyngeal environment. Potential matches were filtered in order to retrieve an unequivocal classification for each read. Full details about this procedure can be found in S1 File.

### Statistical analysis

Differences in demographic parameters were evaluated by t-test using the software Prism (version 5.02; GraphPad Software, San Diego, CA, USA).

Statistical evaluations among alpha-diversity indices were performed by a non-parametric Monte Carlo-based test, through 9,999 random permutations. Differences in abundances of bacterial taxa and functional pathways among experimental groups were analyzed by Mann-Whitney t-test, using MATLAB software (Natick, MA, USA). p-values < 0.05 were considered as significant for each statistical analysis.

### Data availability

Raw reads are available in NCBI Short Read Archive (SRA, http://www.ncbi.nlm.nih.gov/sra) under accession number PRJNA556341.

## Results

### Study population

Initially, our dataset consisted of a total of 91 MSM; 21 samples were, subsequently, excluded due to a low quantity of raw sequencing reads (i.e.: <40,000). Therefore, the final dataset consisted of 70 samples, divided into 2 groups of patients: negative subjects (“no infection”, n = 45), patients with NG pharyngeal infection (“NG”, n = 25).

No difference in the mean age between NG positive and negative subjects was observed (mean age ± standard deviation: 31.6 ± 8.6 vs 32.2 ± 8.7; p = 0.7).

### Pharyngeal microbiota structure characterization

**Fig 1:**
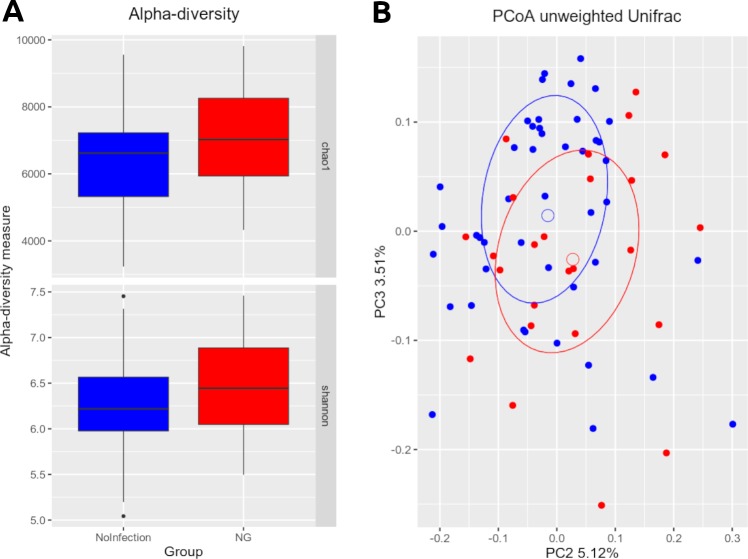
Structure of the pharyngeal microbiota. Microbial composition from pharyngeal swabs of the two groups analyzed: control subjects (No Infection), patients with *N*. *gonorrhoeae* pharyngeal infection (NG). (A) Alpha-diversity boxplots of Chao1 and Shannon index. (B) Principal Coordinates Analysis (PCoA) plot based on unweighted Unifrac distance (beta-diversity). Each point corresponds to a sample from No Infection (blue) and NG (red) groups. For each experimental group, the SEM-based confidence ellipse and the average value centroid are depicted. The second and third principal coordinates are represented.

Alpha-diversity analysis showed no significant differences in biodiversity of infected subjects (Observed Species, p = 0.336; Shannon, p = 0.089; Chao1, p = 0.156; PD_whole_tree, p = 0.417) (Fig 1A). Principal coordinates analysis (PCoA) showed that the microbial profile of infected subjects was not clearly separated from those without NG pharyngeal infections (unweighted Unifrac, p = 0.084; weighted Unifrac, p = 0.333) (Fig 1B).

### Taxonomic composition of pharyngeal bacterial communities

**Fig 2:**
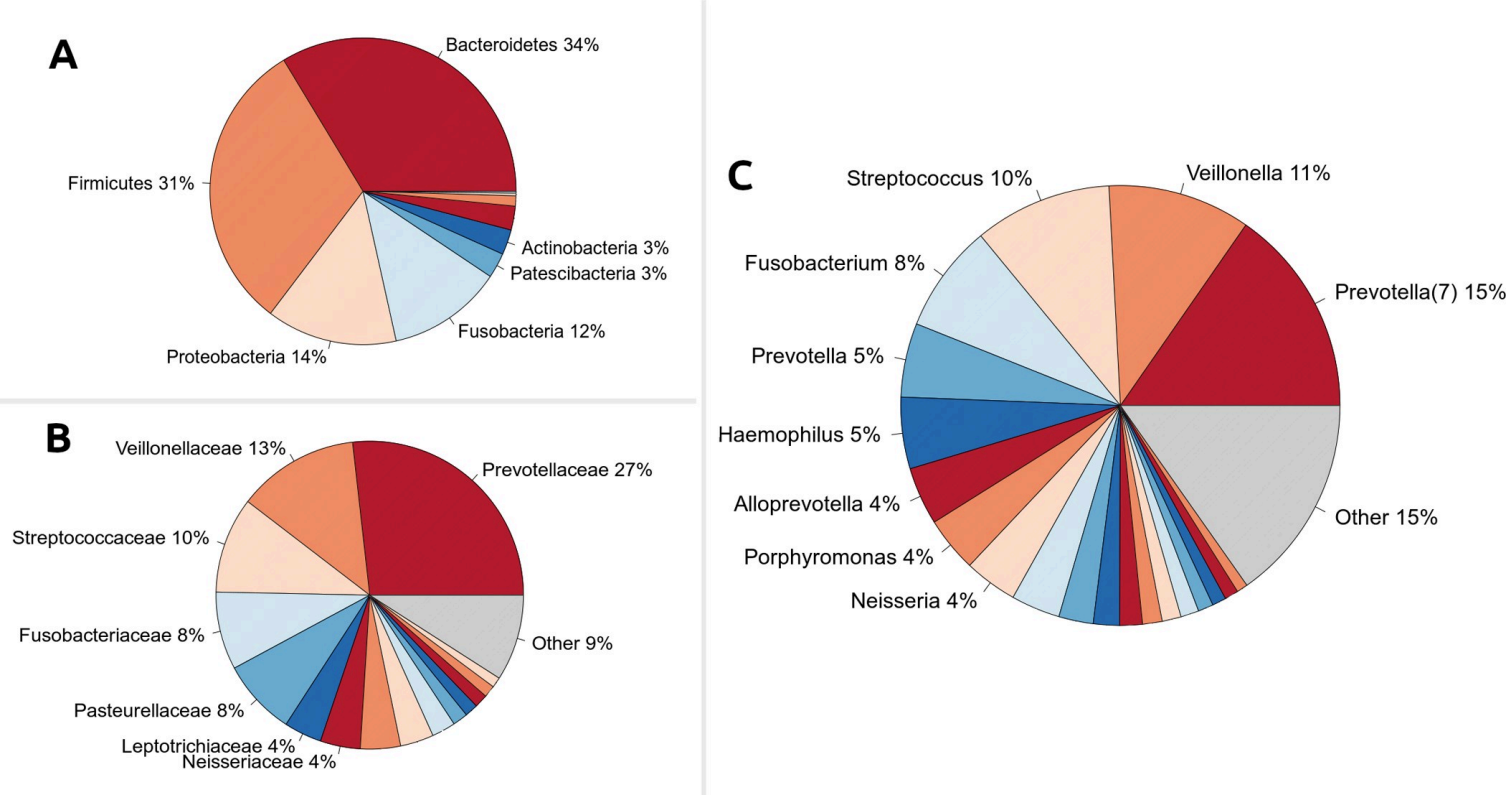
Taxonomic composition of the pharyngeal microbiota. Pie charts of taxonomy relative abundances at (A) phylum, (B) family and (C) genus level for the different subgroups. Only phyla, families and genera present at relative abundances >1% on average in at least one subgroup are reported; remaining taxa are grouped in the “Other” category. Bacterial groups are reported extensively in Table 1 (for phylum, family, genus levels) and Table 2 (for the “Other” genera).

Bacteroidetes, Firmicutes and Proteobacteria dominated the pharyngeal microbiota both in non-infected and NG-positive subjects, with an overall mean relative abundance (mean rel. ab.) in the whole dataset of about 33%, 31% and 14%, respectively. Less represented in the pharyngeal microbiota composition were Fusobacteria (12.1%), Patescibacteria (2.6%) and Actinobacteria (2.6%) (Fig 2A). No significant differences in their abundances across groups were found; however, NG patients were characterized by significantly higher levels of Spirochaetes phylum, compared to non-infected subjects (p = 0.007).

**Table 1:** Relative abundances of the main bacterial groups. Data are expressed as mean ± standard deviation (SD) for phylum, family and genus phylogenetic levels. Only bacterial groups with an average relative abundance >1% in at least one of the two experimental groups are listed; for each, significant p-values of the non-parametric Mann-Whitney test are reported.

Phylum	No Infection	NG	p-value
*Bacteroidetes*	33.67 ± 6.77	33.67 ± 8.63	0.9902
*Firmicutes*	30.91 ± 9.45	31.09 ± 8.47	0.7780
*Proteobacteria*	13.62 ± 8.61	14.02 ± 10.35	0.8734
*Fusobacteria*	11.95 ± 6.69	12.35 ± 5.45	0.8159
*Patescibacteria*	3.09 ± 3.00	2.21 ± 1.82	0.3090
*Actinobacteria*	2.92 ± 2.53	2.37 ± 2.30	0.2493
*Epsilonbacteraeota*	2.66 ± 1.52	2.37 ± 1.37	0.4547
*Spirochaetes*	0.82 ± 1.09	1.34 ± 1.24	0.0075
Other	0.35	0.58	—
**Family**			
*Prevotellaceae*	27.41 ± 7.78	26.16 ± 9.30	0.3516
*Veillonellaceae*	12.86 ± 6.29	12.76 ± 6.33	0.9316
*Streptococcaceae*	10.70 ± 8.73	9.47 ± 6.00	0.9219
*Fusobacteriaceae*	7.49 ± 5.75	8.85 ± 4.82	0.1897
*Pasteurellaceae*	7.26 ± 5.70	8.70 ± 6.70	0.4621
*Leptotrichiaceae*	4.46 ± 3.82	3.49 ± 3.02	0.2297
*Neisseriaceae*	3.72 ± 3.35	4.69 ± 4.65	0.6502
*Porphyromonadaceae*	3.65 ± 3.51	4.79 ± 2.98	0.0607
*Lachnospiraceae*	3.24 ± 1.69	3.79 ± 1.44	0.0862
*Campylobacteraceae*	2.65 ± 1.52	2.34 ± 1.39	0.4115
*Actinomycetaceae*	1.90 ± 2.11	1.27 ± 1.24	0.1698
*Flavobacteriaceae*	1.44 ± 2.39	1.33 ± 2.32	0.6414
*Family XI*	1.19 ± 1.72	1.61 ± 2.10	0.1285
*Saccharimonadaceae*	1.42 ± 1.34	1.07 ± 0.91	0.2543
*Spirochaetaceae*	0.82 ± 1.09	1.34 ± 1.24	0.0075
*Enterobacteriaceae*	1.21 ± 4.67	0.19 ± 0.58	0.1077
Other	8.60	8.13	—
**Genus**			
*Prevotella 7*	15.56 ± 6.09	14.90 ± 7.95	0.6239
*Veillonella*	10.14 ± 5.47	11.18 ± 5.69	0.3032
*Streptococcus*	10.56 ± 8.62	9.38 ± 5.94	0.9025
*Fusobacterium*	7.49 ± 5.75	8.85 ± 4.82	0.1897
*Prevotella*	5.28 ± 2.55	5.73 ± 2.60	0.4185
*Haemophilus*	5.06 ± 4.49	5.61 ± 4.10	0.4256
*Alloprevotella*	4.36 ± 3.06	4.07 ± 2.67	0.8159
*Porphyromonas*	3.65 ± 3.51	4.79 ± 2.98	0.0607
*Neisseria*	3.63 ± 3.32	4.42 ± 4.53	0.7780
*Leptotrichia*	4.14 ± 3.94	2.66 ± 2.66	0.1005
*Campylobacter*	2.65 ± 1.52	2.34 ± 1.39	0.4115
*Actinobacillus*	1.60 ± 3.07	2.51 ± 4.36	0.3977
*Actinomyces*	1.90 ± 2.11	1.26 ± 1.24	0.1623
*Prevotella 6*	1.66 ± 1.49	1.07 ± 1.04	0.0980
*Capnocytophaga*	1.44 ± 2.39	1.33 ± 2.31	0.6414
*Gemella*	1.19 ± 1.72	1.61 ± 2.10	0.1285
*Lachnoanaerobaculum*	1.04 ± 0.80	1.28 ± 0.81	0.1815
*Megasphaera*	1.20 ± 1.06	0.72 ± 0.78	0.0697
*Treponema 2*	0.82 ± 1.09	1.34 ± 1.24	0.0075
Other	16.65	14.95	—

**Table 2:** Bacterial genera of the pharyngeal microbiome showing significant differences in average relative abundance between the groups. Only genera with rel. ab. <1% on average in both experimental groups are shown. Data are expressed as mean ± standard deviation (SD). For each genus, significant p-values of the non-parametric Mann-Whitney test are reported.

Genus	No Infection	NG	p-value
*Catonella*	0.4715 ± 0.3807	0.7572 ± 0.4509	0.0078
*Parvimonas*	0.2197 ± 0.2711	0.4723 ± 0.8314	0.0394
*Filifactor*	0.1933 ± 0.3481	0.4387 ± 0.6166	0.0053
*Selenomonas 3*	0.5430 ± 0.6141	0.2411 ± 0.2879	0.0174
*Uncultured Leptotrichiaceae*	0.0844 ± 0.2518	0.1381 ± 0.3022	0.0163
*Lachnospiraceae (other)*	0.0762 ± 0.0717	0.1352 ± 0.1371	0.0249
*Pseudomonas*	0.8807 ± 3.6333	0.1293 ± 0.2809	0.0054
*Peptococcus*	0.0379 ± 0.0613	0.1130 ± 0.1432	0.0015
*Fretibacterium*	0.0325 ± 0.0518	0.1126 ± 0.1987	0.0007
*Oceanivirga*	0.0199 ± 0.0601	0.1009 ± 0.3667	0.0398
*Ezakiella*	0.0133 ± 0.0283	0.0961 ± 0.1819	0.0013
*Peptoanaerobacter*	0.0502 ± 0.2590	0.0810 ± 0.1798	0.0002
*[Eubacterium] yurii group*	0.0268 ± 0.0483	0.0524 ± 0.0655	0.0376
*Selenomonas 4*	0.0117 ± 0.0168	0.0416 ± 0.1258	0.0488
*Escherichia-Shigella*	0.1253 ± 0.8270	0.0006 ± 0.0015	0.0243

At a lower taxonomic level, *Prevotellaceae* (mean rel. ab. 26.7%), *Veillonellaceae* (12.7%) and *Streptococcaceae* (10.1%) were the most abundant families (Fig 2B). *Spirochaetaceae* were found significantly more relatively abundant in NG-infected patients compared to non-infected ones (p = 0.007).

*Prevotella* was the most represented genus (22.3%), followed by *Streptococcus*, *Veillonella* and *Fusobacterium* (Fig 2C). Patients with pharyngeal gonorrhoea exhibited higher levels of *Treponema* than non-infected individuals (p = 0.007). Taxa abundances for dominant and subdominant constituents (rel. ab >1% in at least one experimental group) of the pharyngeal microbiota at different phylogenetic levels are extensively listed in Table 1.

Considering the less represented genera of the pharyngeal microbiota (rel. ab. <1% on average in both experimental groups), several significant differences were noticed between the two groups (Table 2). An overall imbalance between aerobe and anaerobe genera was observed: the pharyngeal microbiome of NG-infected patients was enriched by several anaerobe bacteria (e.g. *Parvimonas*, *Peptococcus*, *Catonella*, *Filifactor*, *Peptoanaerobacter*) and depleted in various aerobe genera (i.e. *Pseudomonas*, *Escherichia*), compared to non-infected patients. Globally, low abundance genera accounted for about 16% rel. ab. on average.

Notably, although *Neisseria* genus *per se* was found unaltered (mean rel. ab.: 3.6% *vs*. 4.4% in “no infection” and “NG” individuals, respectively), species-level characterization allowed to confirm that non-infected subjects did not carry *N*. *gonorrhoeae*, differently from members of “NG” group; as a matter of fact, only 0.002% on average of all *Neisseria* sequences in “no infection” individuals was classified as *N*. *gonorrhoeae*, compared to 5.8% of “NG” individuals. For the other species identified within the genus (e.g. *N*. *meningitidis*, *N*. *subflava* and *N*. *cinerea*), no substantial differences in the relative abundance were found between the groups. About half of *Neisseria* sequences, however, remained unclassified at species level (S1 Fig).

### Metabolic pathway analysis

Significant differences in several functional pathways were found between NG-infected patients and the control group (Table 3). The microbiome of patients with pharyngeal gonorrhoea exhibited a higher involvement of D-glutamine and D-glutamate metabolism, glycosphingolipid biosynthesis, indole alkaloid biosynthesis and carbohydrate metabolism. In addition, the energy metabolism was observed to be more activated in NG-infected patients compared to non-infected controls.

**Table 3:** Metabolic functional analysis prediction. Main significant KEGG pathways significantly changed between groups and variation among abundances. For each pathway, significant p-values of the non-parametric Mann-Whitney test are reported.

KEGG LEVEL 2	KEGG LEVEL 3	p-value	Variation
Biosynthesis of Other Secondary Metabolites	Indole_alkaloid_biosynthesis	0.0140	↑ in NG
Carbohydrate_Metabolism	—	0.0499	↑ in NG
Energy Metabolism	Sulfur_metabolism	0.0300	↓ in NG
Glycan Biosynthesis and Metabolism	Glycosphingolipid_biosynthesis_ lacto_and_neolacto_series	0.0407	↑ in NG
Lipid Metabolism	Biosynthesis_of_unsaturated_fatty_acids	0.0205	↓ in NG
Metabolism	Energy_metabolism	0.0361	↑ in NG
Metabolism of Other Amino Acids	D-Glutamine_and_D-glutamate_metabolism	0.0419	↑ in NG
Xenobiotics Biodegradation and Metabolism	Polycyclic_aromatic_hydrocarbon_ degradation	0.0340	↑ in NG
Xenobiotics Biodegradation and Metabolism	Caprolactam_degradation	0.0444	↓ in NG
Xenobiotics Biodegradation and Metabolism	Chlorocyclohexane_and_chlorobenzene_ degradation	0.0120	↓ in NG

## Discussion

To the best of our knowledge, no information about the composition of the pharyngeal microbiome during an ongoing *N*. *gonorrhoeae* infection is currently available in scientific literature.

Therefore, in this study, we characterized, for the first time, the pharyngeal bacterial community profiles of NG-positive patients in a well-selected MSM population, excluding several conditions interfering with the oro-pharyngeal environment, and compared them to non-infected individuals.

At first, we noticed that NG-infected patients harboured a pharyngeal microbiome quite similar to negative subjects. This finding goes in the opposite direction from what is usually observed in other microbial niches, such as the vaginal tract. Indeed, during sexually transmitted infections, the vaginal microbiome shifts from a lactobacilli-dominated environment to a dysbiotic condition, characterized by higher levels of anaerobe bacterial genera [20].

Nevertheless, when looking to less-represented bacterial species of the pharyngeal microbiome (rel. ab. of approximately 1% or less), we observed that the oropharynx of NG-positive patients was enriched of various anaerobe bacteria, including *Treponema*, *Parvimonas* and *Peptococcus*, with a contemporary reduction of aerobe genera such as *Pseudomonas* and *Escherichia*.

Even though the exact contribution of these bacterial species should be in-depth analyzed, it is worth mentioning that low abundance microorganisms accounted for about 16% of the total relative abundance. Thus, globally, low-abundance genera could contribute to each other in the creation of tangible differences in a peculiar ecological niche.

Similar changes were recently noticed also in the rectal microbiome during *N*. *gonorrhoeae* infection: NG-positive individuals showed a more marked imbalance between aerobe and anaerobe genera, with lower levels of *Escherichia* species and higher relative abundance of *Peptoniphilus*, *Peptostreptococcus* and *Parvimonas*, compared to non-infected patients [12].

Interestingly, two such different microbial environment, as the pharyngeal and rectal site, can be characterized by similar microbial changes during an ongoing *N*. *gonorrhoeae* infection.

Although we could not determine clearly whether these alterations are caused by gonococcal infection, we can speculate that the oxygen consumption by the pathogen itself or by the recruited leukocytes could promote the proliferation of strict anaerobes. On the other hand, unfortunately, we cannot completely rule out that these microbial changes precede gonococcal infection, favouring NG colonization of the oropharynx.

It has been shown that *Neisseria* species are extremely well adapted to survive in the human nasopharynx, being able to replicate in a nutrition-poor environment and to resist immune and competitive pressure within a polymicrobial complex. Moreover, temperature and relative gas concentrations (nitric oxide and oxygen) found in the nasopharyngeal environment act as potent initial signals for *Neisseria* species adaptation and survival [21, 22].

When colonizing the mucosal epithelia, *N*. *gonorrhoeae* expresses a repertoire of factors that allow replication and survival, as well as modulation and avoidance of the host immune system. After initial adherence, *N*. *gonorrhoeae* replicates and forms microcolonies and, possibly, biofilms, and likely competes with the resident microbiota, in order to effectively persist and meet its nutritional requirements [6].

Previous studies have focused on the interactions between commensals and pathogenic *Neisseria* species of the oropharynx, showing that commensals can compete for niches with pathogens and, thus, provide protection from colonization and invasion [23]. For example, *N*. *lactamica* can displace *N*. *meningitidis* from the nasopharynx and hinder *N*. *meningitidis* acquisition [24]. Moreover, it has been shown that bacteriocins produced by commensal streptococci are bactericidal to *Neisseria* [25].

The persistence of NG in a particular ecological niche depends on various and complex factors: among them, the types and local concentrations of specific nutrients (for example, iron, zinc, glucose, lactate and pyruvate), the oxygen concentration at different sites during colonization, the pattern of cytokine and chemokine responses, and the composition of local microbiota are worthy of note [6, 26]. However, it has not been thoroughly determined which microenvironments are encountered by NG during colonization/infection of the different mucosal epithelia, including the oro-pharyngeal niche.

*N*. *gonorrhoeae* has developed an array of responsive transcriptional factors to activate and repress small- and large-scale adaptive transcriptional programs, in order to survive in specific environmental conditions. As an example, in order to respond to different oxygen concentrations, *N*. *gonorrhoeae* is capable of either aerobic or anaerobic respiration controlled through a truncated denitrification pathway [27, 28].

In this context, the changes in the microbiome composition found in the oropharynx of NG-infected patients (i.e. shift towards higher levels of anaerobes) could be associated with peculiar metabolic variations. Patients with pharyngeal gonorrhoea exhibit a more abundant involvement of D-glutamine and D-glutamate metabolism, and carbohydrate metabolism, as well as a greater activation of the energy metabolism. Although non-predictive further studies are needed for a thorough comprehension of the pharyngeal metabolic changes during gonorrhoea, we can speculate that the shifts towards anaerobe bacteria is accompanied by a higher activation of the energy metabolism, with higher metabolism of carbohydrates and amino acids. This, in turn, could lead to the production of metabolites that contributes to NG growth and proliferation in the oropharynx.

As an example, carbohydrate consumption by anaerobe bacteria can result in the production of several metabolites, including lactate and other organic acids. In this context, it has been shown that lactate utilization permits survival of *Neisseria* species in nasopharyngeal tissue [29]. Similarly, propionic acid is used by *Neisseria* species as a growth substrate, thus contributing to their abundance in the upper respiratory tract [30].

We are fully aware of some limitations of this study. At first, more exhaustive information about patients (e.g. time between *N*. *gonorrhoeae* exposure and sampling; number of sexual partners; more in-depth data about oral health) would have been useful to find deeper correlations between clinical/behavioral factors and microbiome alterations.

Moreover, additional in-vivo and in-vitro studies will be needed to investigate the role and the contribution of less-represented species of the pharyngeal environment in terms of metabolic activity and inter-species competition.

Finally, further prospective investigations, including a larger number of subjects, will be essential to confirm our findings and to understand if the alterations of the pharyngeal microbiome precede or follow gonococcal infections.

In conclusion, although preliminary, our data could shed light on the complex microbial dynamics occurring during *N*. *gonorrhoeae* colonization of the pharyngeal microenvironment. In this way, it will be possible to set up new innovative strategies for the control and prevention of pharyngeal gonorrhoea.

## Supporting information

S1 File Detailed methods about species-level classification of *Neisseria* genus. (PDF)

S1 Fig Barplots of average relative abundances of the *Neisseria* species found in the samples. Only the five most abundant were represented, whereas all others are represented in the ‘Other *Neisseria* sp.’ group. Relative abundances refer to the proportion of each species among all reads classified in the *Neisseria* genus.
(TIFF)
