# Supplementary material for: Pharyngeal microbiome alterations during Neisseria gonorrhoeae infection
Source: PLoS One. 2020 Jan 16;15(1):e0227985. doi: 10.1371/journal.pone.0227985 (PMC6984747; doi:10.1371/journal.pone.0227985)
Supplement: S1 File — (PDF) [file pone.0227985.s001.pdf]

Species-level analysis of Neisseria genus

Classification of reads belonging to *Neisseria* was further improved, where possible, down to the species level, via a BLAST-based [**S1**] re-classification on an *ad-hoc* built reference database.

Reference sequences

Due to the ubiquitarian presence of *Neisseria* species in different environments, we limited the re-analysis to the 12 most frequently found species in the oro-pharyngean environment, downloading full genome sequences from NCBI RefSeq database for bacteria (ftp://ftp.ncbi.nlm.nih.gov/genomes/refseq/bacteria/), for a grand total of 985 strains.

Through a custom script, sequenced genomes for all species and strains were downloaded and properly formatted for further processing. In all our analyses, only bacterial strains with a genome finishing grade of “Complete”, “Chromosome” or “Scaffolds” were considered. The following table summarizes the references used for each species.

Species name	Number of strains
Neisseria cinerea	1
Neisseria elongata	7
Neisseria flavescens	1
Neisseria gonorrhoeae	210
Neisseria lactamica	6
Neisseria macacae	1
Neisseria meningitidis	749
Neisseria mucosa	2
Neisseria perflava	2
Neisseria polysaccharea	2
Neisseria sicca	2
Neisseria subflava	2

Reads to re-classify

From the OTU table comprising all the samples, OTUs classified within the *Neisseria* genus were selected and the sequences of all the reads grouped in each OTU (clustered at 97% similarity) were retrieved. In order to reduce the number of sequences to re-classify, clonal reads (i.e.: reads being identical throughout 100% of their length and composition) were grouped together.

Classification

Re-classification of the reads was performed through nucleotide BLAST (legacy BLAST, v 2.26), using a cutoff of 1e-10 for the e-value and de-activating the dust-filter. Only reads matching for at least of 80% of their length were retained and, for each read, the best match (i.e.: that or those with the higher bit-score) was selected. If a read had multiple classifications on different species, the classification was reset to genus level.

Statistical analysis

In order to keep only consistent data for species-level evaluations, only samples having a relative abundance of *Neisseria* genus higher than 0.5%, were considered. This was made to exclude samples with very few reads classified in the genus that could profoundly alter the dataset (e.g.: considering a sample in which we had only 1 read in a genus, this would have brought a 100% to the species-level classification for that certain species). Since the least sequenced sample had about 36800 reads, this equaled having at least 180 reads in Neisseria genus; 59 out of the 70 samples were, thus, considered. A non-parametric Mann-Whitney U-test was used to compare the relative abundance of each bacterial species in the different experimental groups, considering p-values <0.05 as significant. Statistical evaluations were carried out using Matlab (v 2008b, Natick, MA, USA)
