# Supplementary figures and images for: Pharyngeal microbiome alterations during Neisseria gonorrhoeae infection
Source: PLoS One. 2020 Jan 16;15(1):e0227985. doi: 10.1371/journal.pone.0227985 (PMC6984747; doi:10.1371/journal.pone.0227985)

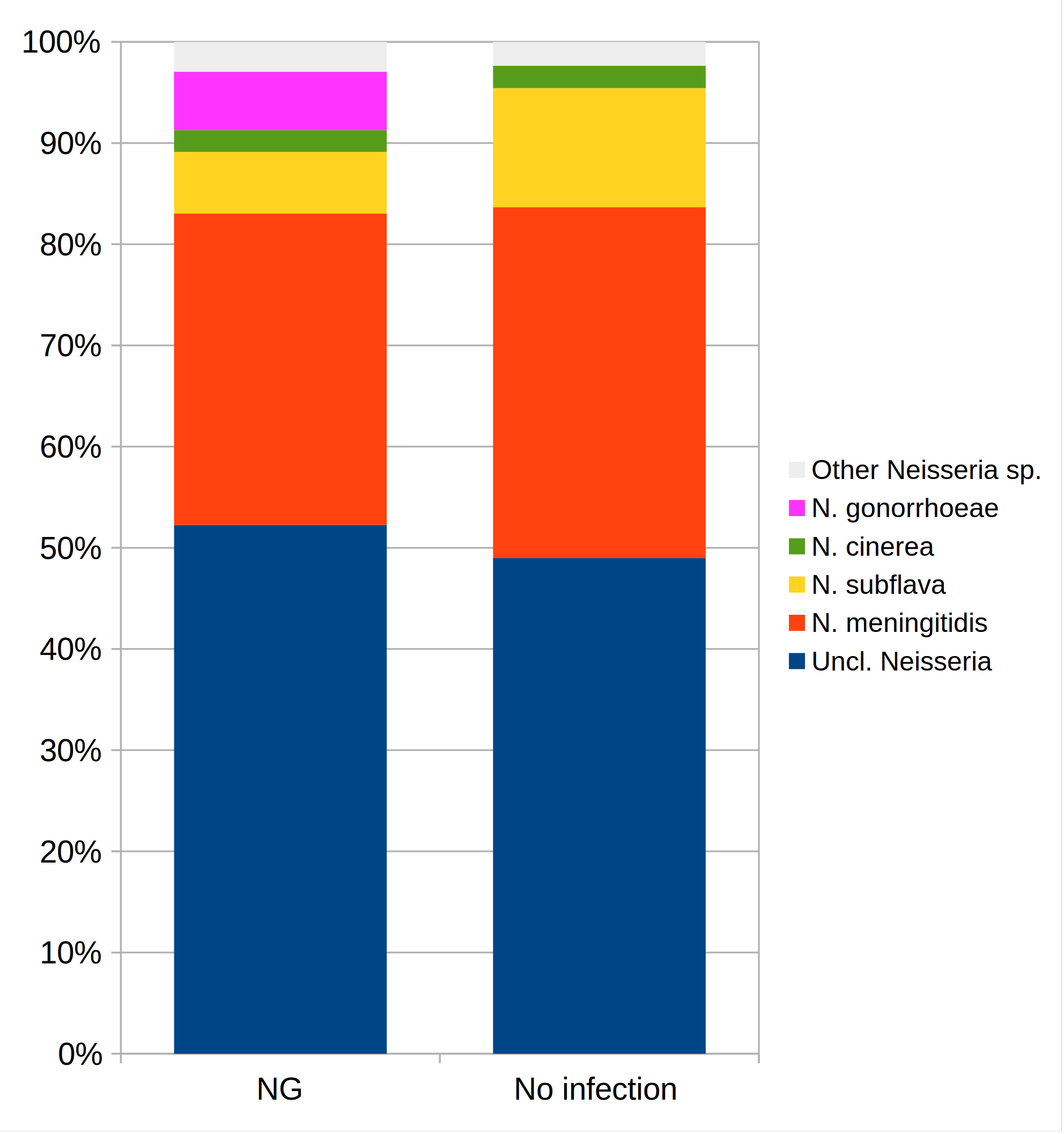

Supplement: S1 Fig — Only the five most abundant were represented, whereas all others are represented in the ‘Other Neisseria sp.’ group. Relative abundances refer to the proportion of each species among all reads classified in the Neisseria genus. (TIFF) [file pone.0227985.s002.tiff]
